# Deep sequencing-derived Metagenome Assembled Genomes from the gut microbiome of liver transplant patients

**DOI:** 10.1038/s41597-024-04153-8

**Published:** 2025-01-09

**Authors:** Goutam Banerjee, Suraya Rahman Papri, Hai Huang, Sanjaya Kumar Satapathy, Pratik Banerjee

**Affiliations:** 1 Department of Food Science and Human Nutrition, University of Illinois at Urbana-Champaign, Urbana, IL 61801 USA; 2 The Feinstein Institutes for Medical Research, Northwell Health, Manhasset, NY USA; 3 Division of Hepatology, Sandra Atlas Bass Center for Liver Diseases & Transplantation, Donald and Barbara Zucker School of Medicine at Hofstra/Northwell Health, Manhasset, NY USA

**Keywords:** Non-alcoholic fatty liver disease, Metagenomics

## Abstract

Recurrence of metabolic dysfunction-associated steatotic liver disease (MASLD) after liver transplantation (LT) is a continuing concern. The role of gut microbiome dysbiosis in MASLD initiation and progression has been well established. However, there is a lack of comprehensive gut microbiome shotgun sequence data for patients experiencing MASLD recurrence after LT. In this data descriptor, we describe a dataset of deep metagenomic sequences of a well-defined LT recipient population. Community-based analysis revealed a high abundance of *Akkermansia muciniphila*, consistently observed in most patient samples with a low (0–2) MASLD Activity Score (NAS). We constructed 357 metagenome-assembled genomes (MAGs), including 220 high-quality MAGs (>90% completion). The abundance of different species of *Bacteroides* MAGs dominated in patient samples with NAS > 5 (“definite MASH”). In contrast, the MAGs of *A. muciniphila*, *Akkermansia* sp., and *Blutia* sp. dominated in samples from patients without MASH (NAS = 0–2). In addition, the phylogenetic analysis of *A. muciniphila* and *Akkermansia* sp. MAGs identified two new phylogroups of *Akkermansia* that are distinct from the previously reported three phylogroups.

## Background & Summary

Metabolic dysfunction-associated steatotic liver disease (MASLD), formerly known as nonalcoholic fatty liver disease (NAFLD), encompasses a wide range of liver disorders, and its escalating prevalence has become a global concern^1^. Estimates indicate that the prevalence of MASLD was approximately 25.5% in 2005, which increased to 38.7% in 2016^2^. The disruption of the gut-liver axis due to an imbalance in the gut microbial community can have a negative impact on energy homeostasis, leading to the development of various metabolic syndromes such as obesity and MASLD^3,4^. Intestinal health is a crucial aspect of MASLD, and consequently, various studies have assessed the makeup of the gut microbial community and its abundance using sequence-based metagenomic approaches^3–6^. For microbiome analysis, deep shotgun sequencing (with more than 10 million reads/sample) provides several advantages over shallow sequencing (<10 million reads/sample) and 16S rRNA amplicon sequencing-based approaches, such as identifying rare microbial taxa (at species levels), classification of uncultivated bacteria, metabolic profiling, host-microbe interactions, novel gene discovery, identification of gene clusters responsible for secondary metabolite production, and for constructing metagenome-assembled genomes (MAGs)^7–9^. The significance of gut microbiota composition and its functions in MASLD warrants the use of deep shotgun metagenomic sequencing. To the best of our knowledge, as of the current time, there is no publicly available ultra-deep shotgun sequencing dataset (with sequencing depth >20 million reads/sample) from patients who have undergone liver transplantation (LT) and subsequently developed MASLD recurrence.

**Fig. 1:**
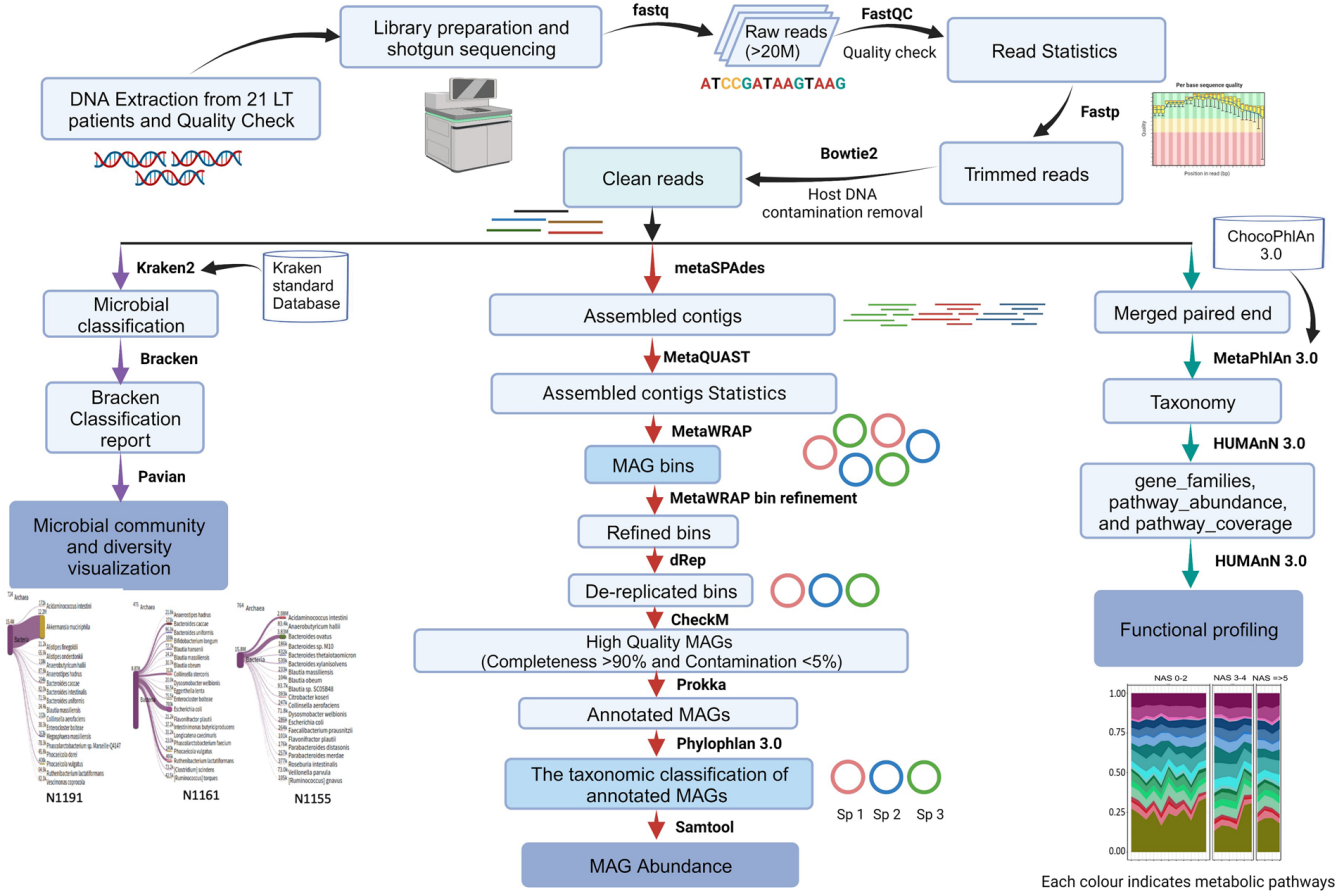
The detail workflow from DNA extraction to bioinformatics analysis. Every step and their associated software packages are given for better understanding of the analysis.

In our previous prospective observational study, the gut microbial community status of LT patients with varying pathologies of metabolic dysfunction-associated steatohepatitis (MASH), formerly known as nonalcoholic steatohepatitis (NASH), recurrence was reported by utilizing the 16S rRNA amplicon sequencing-based approach^4^. As mentioned before, data generated from deep shotgun sequencing of the metagenomic samples is essential for comprehensive community-based functional analyses and constructing draft MAGs, enabling a deeper understanding of the disease outcomes^10^. In this study, we employed a deep shotgun sequencing approach (that generated over 20 million reads per sample) to investigate the gut microbial flora and construct MAGs from liver transplant (LT) patients manifesting varying degrees of MASH recurrence, as illustrated in Fig. 1.

**Fig. 2:**
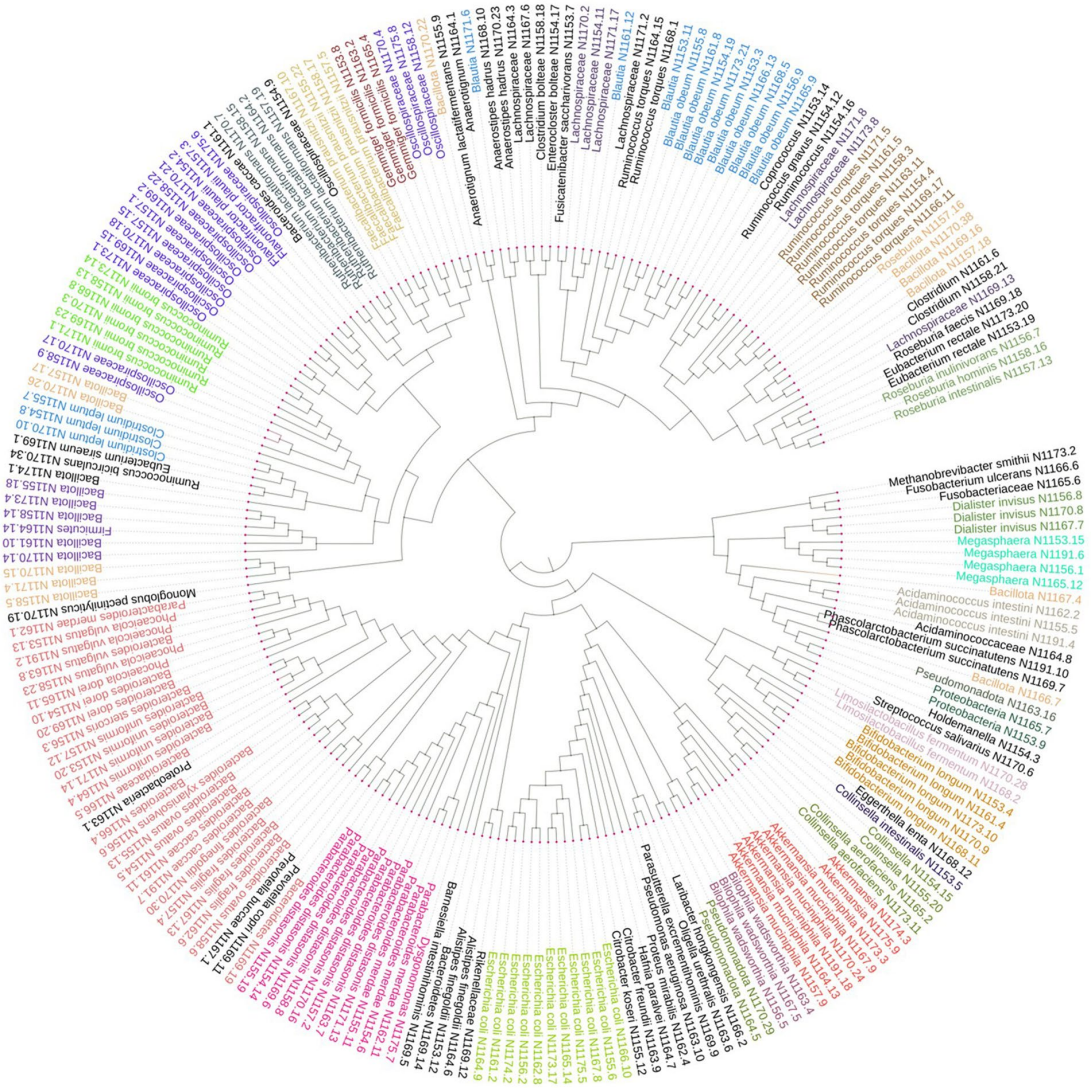
The phylogenetic tree of high-quality MAGs constructed using maximum likelihood method (completeness >90% and contamination <5%) prepared by PhyloPhlAn 3.0 and visualized by iTOL. The different colors in the tree represented a separate cluster of MAGs and their close relatives. The red colored cluster shows the *A. muciniphila* group.

Based on the NAS score, all samples have been categorized into three groups according to conventional clinical practices^11,12^: “no MASH” (NAS 0–2), “borderline MASH” (NAS 3-4), and “definite MASH” (NAS ≥ 5) samples. Patient-level demographic and clinical data at the time of stool sample collection are provided in Table S1. At the phylum level, we observed variations in the abundance of three phyla — Fusobacteria, Euryarchaeota, and Verrucomicrobiota — across these three sample groups. Remarkably, our findings align with our previous research involving 16S rRNA sequencing of the same samples. In this study, we observed a substantial increase in the abundance of *A. muciniphila* and *Akkermansia* sp. in the majority of samples from patients with low NAS (0–2), reaffirming our earlier observations^4^. The species-level functional profiling indicated that the elevated abundance of three amino acid biosynthesis pathways positively correlates with samples from patients with no MASH outcomes [NAS (0–2)]^13,14^. Additionally, we constructed and taxonomically classified the MAGs from all these samples (Fig. 2) and estimated their abundance using a mapping-based approach. The abundance of MAGs of *A. muciniphila* and *Blautia* sp. were very high in most patient samples with low or no MASH activities [NAS (0–2)]. We also have analyzed and compared the MAGs of *A. muciniphila* and *Akkermansia* sp. to explore the phylogenetic groups. This exploration led to identifying two potentially new phylogenetic clusters within *A. muciniphila* and *Akkermansia* sp. The information regarding pathway abundance, in conjunction with the MAGs, may contribute significantly to enhancing our understanding of the underlying disease mechanisms during the progression of MASLD.

## Methods

### Study location, ethical clearance, and sample collection

The study was conducted at James D. Eason Transplant Institute of Methodist University Hospital, affiliated with the University of Tennessee Health Sciences Center, Memphis, TN. Adult LT recipients (age >18) with MASH as an indication for LT, who had a liver biopsy one-year post-transplant, were recruited for this study^4^. The signed consent of all the participants had been taken prior to enrollment in the study. The protocol and the study design were approved by the University of Tennessee Institutional Review Board (Study Protocol # 15-03891-XP UM). The stool samples from each participant were collected in accordance with the specified methodology described previously^4^.

### Fecal microbiota DNA extraction and quality check

The genomic DNA was extracted following the protocol described in the PowerFecal DNA extraction kit (MO BIO Laboratories, Carlsbad, CA). Initially, the quality and the quantity of the extracted DNA were checked on 0.8% agarose gel and NanoDrop spectrophotometer (Thermo Scientific, Wilmington, DE), respectively.

### Shotgun library preparation and sequencing

The library preparation for shotgun sequencing was done following the protocol of the Kapa Hyper Stranded kit (Roche). Subsequently, the quality of the prepared libraries was assessed using the 5200 Fragment Analyzer (Agilent Technologies, USA). The libraries were then pooled; quantitated by qPCR and subjected to sequencing (NovaSeq. 6000) on one SP lane for 151 cycles from both ends of the fragments. The sequencing was done using paired-end reads (2 × 150 bp), yielding more than 25 million reads per sample on average, ensuring a robust coverage for subsequent analysis and interpretation of the genomic data.

### Host contamination removal

To enhance the accuracy of the downstream analysis, it is crucial to remove host DNA contamination from metagenomic reads. Here, we employed a mapping based method using bowtie2 v2.5.0^15^ to remove human DNA contamination. In brief, *bowtie2 index* command was used to index the human reference genome (GRCh38) obtained from the NCBI database (https://www.ncbi.nlm.nih.gov/data-hub/genome/GCF_000001405.26/), followed by an alignment step. Any reads that were successfully mapped to the human genome were identified as host DNA and subsequently removed from the dataset.

### Microbial diversity and community analysis

**Fig. 3:**
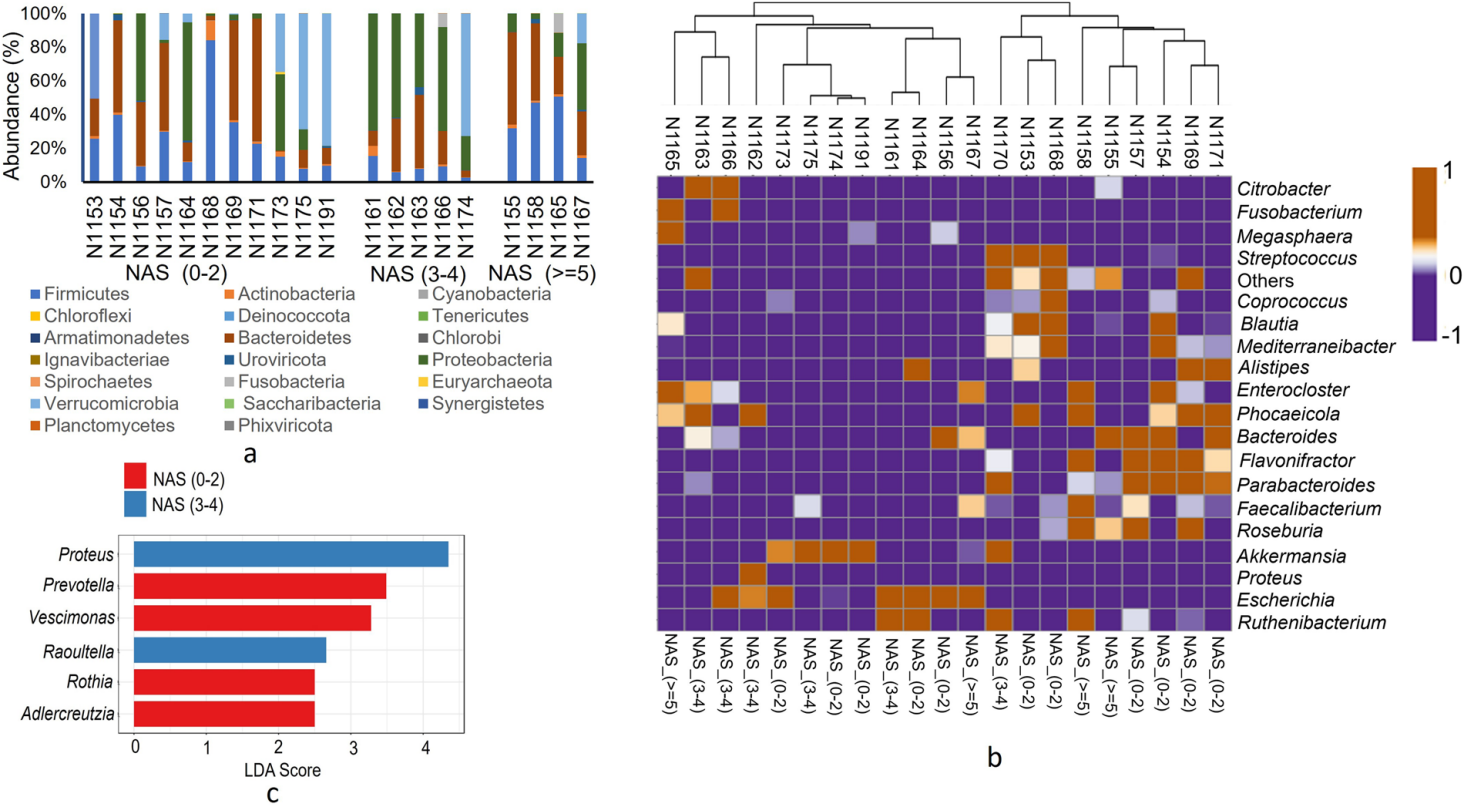
Bracken report based microbial diversity and community in each sample. (**a**) The distribution and abundance of top 20 phyla based on sample groups (**b**) heatmap representing the relative abundance of top 20 genera in different MASLD samples. (**c**) LDA enrichment of significant genera in different MASLD groups calculated using LEfSe considering g p < 0.05 and LDA score 2.0.

The clean microbial reads obtained from bowtie2 were subjected to assign taxonomic composition using Kraken2 package v2.0.8^16^. The mapping of clean reads was done against Kraken2 standard database (https://benlangmead.github.io/aws-indexes/k2) (accessed on 11/02/2022). The output file (Kraken.report) generated in Kraken2 was further used as input in Bracken v2.8^17^ to produce accurate phylum and species level abundance (Fig. 3). Results indicated that Firmicutes (also known as Bacillota) and Bacteroidetes were the most abundant phyla across all samples (Fig. 3a). Interestingly, our analysis revealed notable differences in the presence of the Euryarchaeota and Verrucomicrobia phyla among the sample groups. Specifically, these phyla were detected in the majority of “no MASH” (NAS 0–2) samples but were largely absent in the “borderline MASH” (NAS 3-4), and “definite MASH” (NAS ≥ 5) samples (Fig. S1). Furthermore, the relative abundance at the genus level was calculated, and the top 20 genera are presented in Fig. 3b. The results indicated that the genus *Akkermansia* was highly abundant in samples from the NAS 0–2 and NAS 3-4 groups, with abundances ranging from 0.43 to 0.79 (Fig. 3b). However, in patients with NAS ≥ 5, the abundance of *Akkermansia* was either low or absent in most samples. *A. muciniphila* is generally considered a next-generation probiotic, and its high abundance in the gut is associated with various positive health outcomes, including MASLD^18^. To identify the differential abundance of the key genera in the MASLD sample groups, we performed Linear Discriminant Analysis (LDA) using LEfSe^19,20^ with a threshold of p < 0.05 and an LDA score of 2.0. Although the abundance of *Akkermansia* was high in samples from the NAS 0–2 and NAS 3-4 groups, it was not significantly enriched in these groups., and thus its abundance cannot be correlated with MAFLD outcome. The results indicated that the genera *Proteus* (LDA 4.35) and *Raoultella* (LDA 2.66) were enriched in the NAS 3-4 group (Fig. 3c). In contrast, the genera *Prevotella* (LDA 3.49) and *Vescimonas* (LDA 3.28) were enriched in the NAS 0–2 group. Interestingly, no significant differential effects were observed in the NAS ≥ 5 group.

**Fig. 4:**
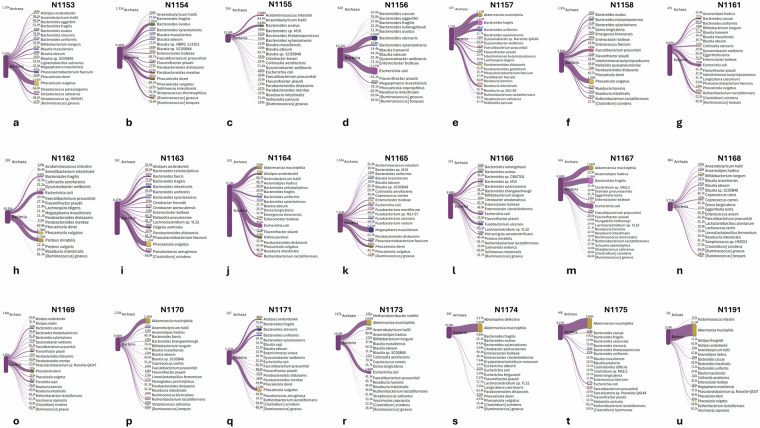
The snakey diagram of the top 20 species generated from the Pavian platform and their absolute abundance in terms of read counts.

Species level diversity was further analyzed and visualized using the Pavian platform through Sankey flow diagrams (Fig. 4)^21^. The high abundance of *A. muciniphila* was consistently observed in samples from patients without MASH outcomes [NAS (0–2)] (Fig. 4r,t,u).

Furthermore, the alpha diversity indices (Table S2) strongly support our diversity findings. For example, sample N1191 (Fig. 4u) is highly dominated by a single species *A. muciniphila*, and thus its overall species diversity is low which is indicated by high Berger Parker’s dominance value (0.78571) and lower Simpson’s index value (0.3798).

### Functional profiling of gut microbial community

**Fig. 5:**
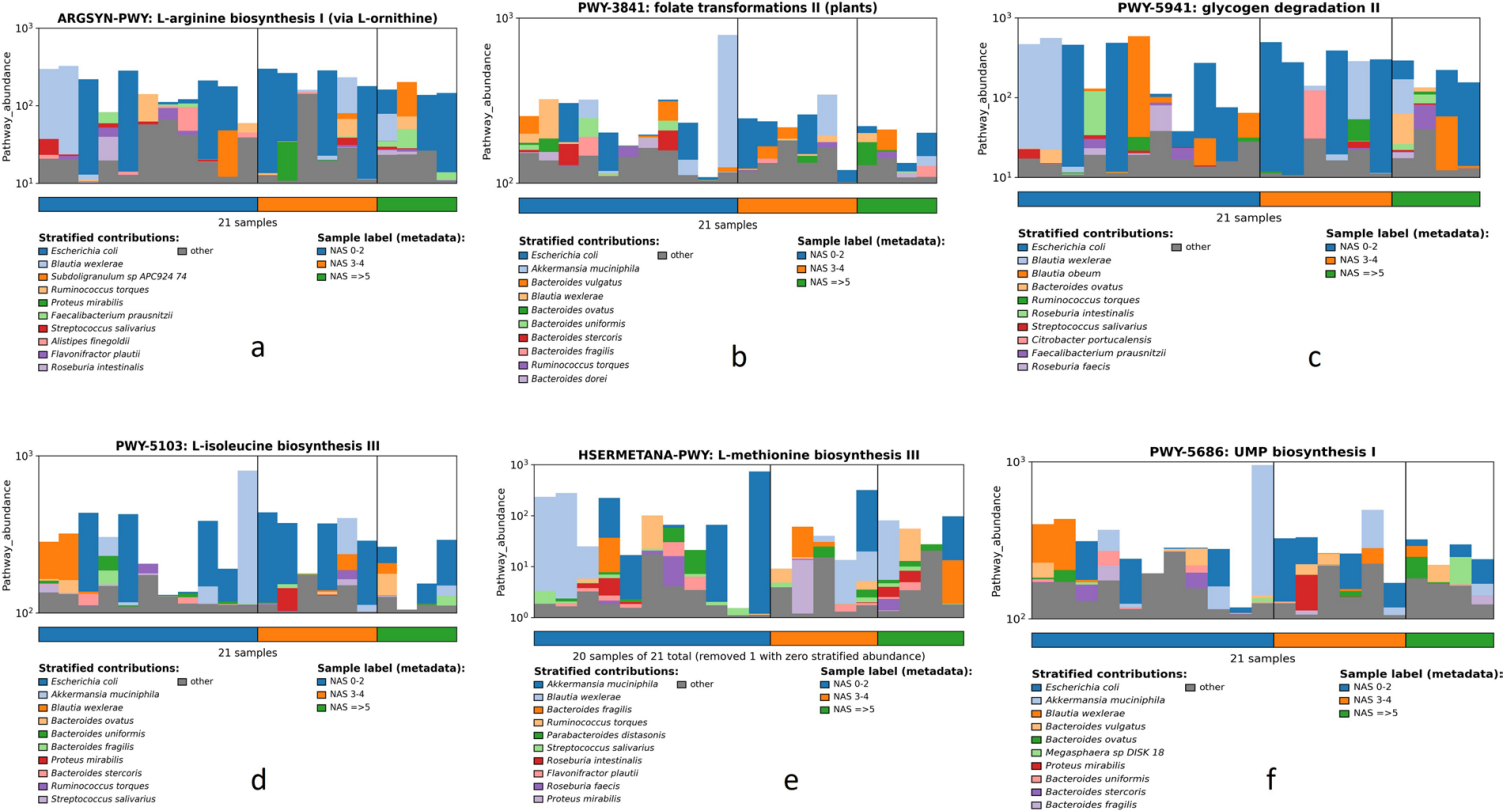
The functional profile of the top 10 species in each sample. (**a**-**f**) Here we have demonstrated six important pathways that might directly correlate with MASLD outcome. The important pathways were either dominated by *A. muciniphila* or *Blautia* sp.

Functional profiling was done to gain a deeper understanding of the functional potential of the microbial communities and how it may relate to the observed pathologies associated with MASLD progression. In brief, the paired-end clean reads of each sample (r1 and r2) were first merged with *Cat* command in the Linux platform. The merged file was then used in MetaPhlAn v4.0^22^ to generate the taxonomy file. The merged clean read file and the corresponding taxonomy file were used as input in HUMAnN v3.0^23^ to generate three files: gene_families, pathway_abundance, and pathway_coverage using *ChocoPhlAn* database v201901b (accessed on 04/23/2023). All pathway_abundance files obtained from 21 samples were merged, normalized in HUMAnN v3.0 using *humann_renorm_table* command, and plotted using *humann_barplot* command (Fig. 5). Several pathways like the folate transformations (Fig. 5b), L-isoleucine biosynthesis (Fig. 5d), and L-methionine biosynthesis (Fig. 5e) exhibited higher abundance in “no MASH” (NAS 0–2) samples compared to “definite MASH” (NAS ≥ 5) samples, and interestingly, all these pathways were dominated by *A. muciniphila*. Similarly, the abundance of the other two microbial pathways: L-arginine biosynthesis (Fig. 5a) and glycogen degradation (Fig. 5c) were also high in low NAS (NAS 0–2) samples compared simples with NAS ≥ 5 (definite MASH), and dominated by the probiotic bacteria *Blautia* sp^14^. Previous research highlighted that an abundance of these pathways are associated with normal liver function^13,14,24–26^.

### Metagenomic assembly, contig generation, and quality check

Metagenome assembly is the first step in metagenome assembled genome (MAG) construction^27^. Here, we used SPAdes v3.15.5^28^ to construct long contigs from the clean reads using a *de novo* approach using *–meta* option. The quality and length of these assembled contigs were assessed by MetaQUAST^29^.

### Binning and refinement of MAGs

Binning is the most critical step in the construction of MAG and here we used MetaWRAP^30^ for binning the contigs obtained from metaSPAdes. MetaWRAP is a wrapper of three binning packages: MaxBin2, MetaBAT2, and CONCOCT. The bins obtained from MetaWRAP were often fragmented due to uneven coverage and inter-species overlapping; thus, bin refinement is also recommended. The *metawrap bin_refinement* command was used to refine the bins generated from MaxBin2, MetaBAT2, and CONCOCT using -c 50 -x 5 option, which has generated a total of 357 draft genomes or MAGs.

### Completeness, contamination, and taxonomy of MAGs

**Fig. 6:**
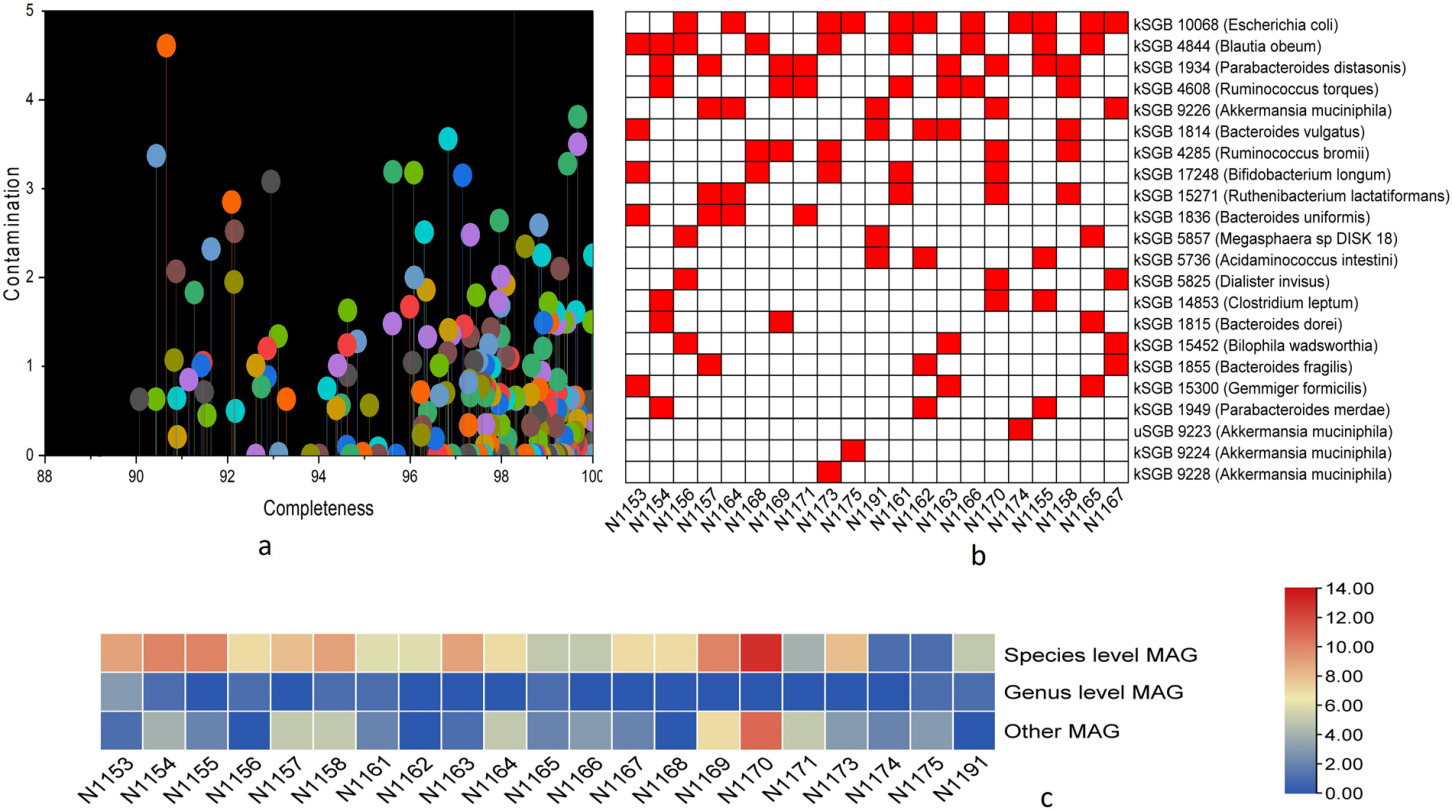
The distribution of meta genome assembled genome (MAGs) in each sample. (**a**) Indicated the completeness and contamination of 220 high-quality MAGs. Each color represented a single MAG, and the attached line indicated the percentage of contamination. (**b**) Represent the top known species level genome bins or MAGs in different samples. Here, red and white blocks mean the presence and absence, respectively. (**c**) Along with species-level MAGs, several genus-level and other lineage MAGs were also constructed and presented here. The color density bar on the right side indicated the number of specific level MAGs in different samples.

The completeness and contamination of MAGs were further assessed by using CheckM v1.1.3^31^, and the result of high-quality MAGs (>90% completeness and <5% contamination) was tabulated accordingly (Table S3, Fig. 6a). We have documented the most abundant species-level MAGs, providing insights into the specific microbial species that are abundant within the samples (Fig. 6b). The overall distribution of high-quality MAGs, categorizing them at different taxonomic levels, including species, genus, and other taxonomic ranks was illustrated in Fig. 6c. The high-quality MAGs were annotated with Prokka v1.14.6^32^ using a default command. The taxonomic classification of annotated MAGs was performed in Phylophlan 3.0^33^, and the generated tree file was visualized in iTOL online platform (https://itol.embl.de/) (Fig. 2).

### Abundance of MAG

The abundance of each MAG in respective samples was calculated following the method described by Zorrilla *et al*.^34^. In brief, fasta files of each MAGs generated in Prokka were merged (each sample separately) using *Cat* command, followed by mapping in bwa v0.7.17^35^ to generate sam files. The *Samtool_view* and *samtools_ sort* commands^36^ were used to convert the sam file to the sorted bam file. The *samtools flagstat* command was finally employed to calculate the mapping reads, and the relative abundance of each MAG was calculated as the total number of mapped reads divided by the total number of reads in the corresponding sample (Table S4). Interestingly, in all “definite MASH” samples (NAS ≥ 5) except one sample (N1167), the dominant species level MAGs belong to *Bacteroides ovatus* (N1155, 36.58%), and *Bacteroides vulgatus* (N1158, 15.11%), and *Bacteroides dorei* (N1165, 10.8%) (Table S4). However, in most of the samples from patients without MASLD or MASH (NAS 0–2) the abundant MAGs belong to the species *Blautia obeum* (ranges between 12.41% to 41.46%)), *A. muciniphila* (ranges between 25.31% to 72.49%) (and *Akkermansia* sp. (54.38%) (Table S4).

### Average nucleotide identity and phylogenetic analysis of *Akkermansia* MAGs

**Fig. 7:**
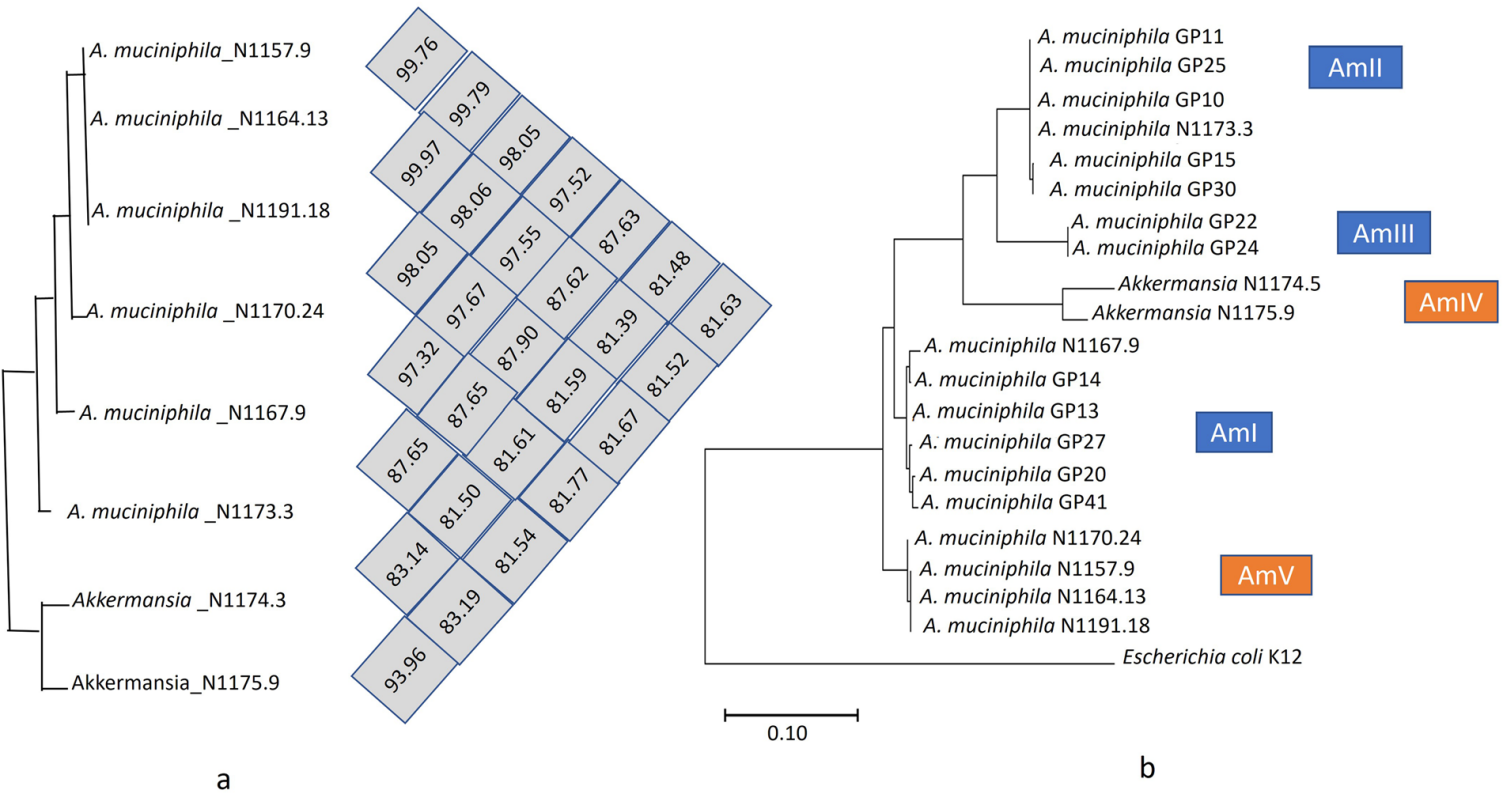
The comparison of *A. muciniphila* MAGs constructed from different samples. (**a**) Represented the average nucleotide identity among different MAGs. (**b**) demonstrate the phylogenetic tree of *A. muciniphila* showing different phylogenetic groups. *Escherichia coli* was taken as an out-group.

Most of the “no MASH” (NAS 0–2) samples have a high abundance of *A. muciniphila*, which indicates a positive correlation with MASLD status. Therefore, we calculated the average nucleotide identity (ANI) among these *A. muciniphila* and *Akkermansia* sp. MAGs using OrthoANI tool v0.93.1^37^ (Fig. 7a). Interestingly*, A. muciniphila* MAGs obtained from samples with low NAS (0–2) such as AK_N1157.9, AK_N1164.13, AK_N1191.18 clustered together which indicates that the *A. muciniphila* strains from different samples with favorable NAS scores share a high degree of genomic similarity. In order to check the phylogenetic group of the constructed *A. muciniphila* MAGs, we selected a few *A. muciniphila* strains randomly representing different phylogenetic clusters (AmI, AmII, and AmIII)^38^. The fasta files of these strains were downloaded from the NCBI genome database, followed by annotation in Prokka using the default command. The 16S rRNA gene sequences were then extracted and aligned in MEGA 6 software^39^ to generate the phylogenetic tree to compare the phylogenetic position of *A. muciniphila* MAGs and *Akkermansia* sp. MAGs (Fig. 7b). *A. muciniphila* MAG obtained from the N1173 sample (NAS 0) and N1167 (NAS 6) clustered with AmII and AmI, respectively. Furthermore, *A. muciniphila* MAGs constructed from “borderline MASH” (NAS 3–4) sample (N1157) and “no MASH” (NAS 0–2) samples (N1164, N1170, and N1191) clustered together and formed a new phylogroup AMV (Fig. 6B). However, genus level *Akkermansia* MAGs obtained from N1174 and N1175 exhibited an new phylogroup AMIV, along with previously described phylogenetic groups (AmI, AmII, AmIII)^38^. This phylogenetic analysis can shed light on the genetic diversity and adaptation strategies of *A. muciniphila* within the context of MASLD and its correlation with disease outcomes.

## Data Records

The Illumina NovaSeq sequencing reads are available in the NCBI Sequence Read Archive (SRA) under BioProject identifier PRJNA970820^40^, with accession number SRP438221^41^. High quality MAGs (n = 220) are available at SAMN36703611- SAMN36703829 and SAMN36726309 under the same BioProject identifier^40^. The information regarding patient fat percentage and NAS score (Supplementary Table 1), microbial alpha diversity (Supplementary Table 2), MAG quality assessment (Supplementary Table 3), MAG abundance in each sample set (Supplementary Table 4), and differential abundance of three phyla in all sample sets (Supplementary Figure 1) were deposited to figshare^42^ with 10.6084/m9.figshare.27730911.

## Technical Validation

Here, we have explored the microbial diversity and abundance of MAGs in stool samples of LT patients using deep shotgun Illumina sequencing. Microbial community assessment and construction of MAGs underwent a series of quality control processes, including removing host contamination (Fig. 1, Table 1). The sequencing platform generated a total of 538.6 million reads. Following quality filtering with a threshold of q < 25, 535.1 million reads were retained (Table 1). This stringent quality filtering process ensures that only high-quality reads are included in downstream analyses, enhancing the reliability and accuracy of the results obtained from the sequencing data. The mapping percentage of classified clean reads of most of the sample is above 60% (Table 2), confirming the reading quality and depth.

**Table 1:** Sequencing reads processing statistics of each data set based on FastQC result.

Sample ID	Raw reads count	Trimmed reads (q > 25)	% removal after initial trimming	Human DNA removed (clean reads)	% removal of host contamination
N1153	25424028	25281010	0.562530847	25026660	1.006091
N1154	34717232	34525330	0.552757201	34431878	0.270677
N1155	41353794	41044262	0.748497224	40816658	0.554533
N1156	25341390	25187138	0.608695892	25184750	0.009481
N1157	20468100	20339394	0.62881264	20334194	0.025566
N1158	23389134	23251812	0.587118788	23212412	0.169449
N1161	21739642	21568308	0.788117854	21543746	0.11388
N1162	23113510	22965722	0.639400939	22962290	0.014944
N1163	20194568	20065904	0.637121824	20061580	0.021549
N1164	29329826	29179320	0.513149993	29171336	0.027362
N1165	25934688	25766786	0.6474032	24149980	6.274768
N1166	20228664	20118430	0.544939597	20103872	0.072362
N1167	21321082	21191406	0.608205531	21171620	0.093368
N1168	19026784	18924362	0.538304319	18840866	0.441209
N1169	18455136	18339154	0.628453781	18334706	0.024254
N1170	34380902	34123660	0.7482119	34102748	0.061283
N1171	22904742	22745032	0.697279192	22723724	0.093682
N1173	30135136	29913668	0.734916212	29790838	0.410615
N1174	25592112	25417010	0.684203008	25414844	0.008522
N1175	20839126	20723470	0.554994485	20681918	0.200507
N1191	34750680	34520478	0.662438836	34492538	0.080937

**Table 2:** Mapping percentage of clean reads using Kraken 2 standard database.

Sample ID	Classified reads (%)	Microbial reads (%)	Bacterial reads (%)
N1153	61.3	61.3	60.8
N1154	56.8	56.8	54.8
N1155	77.6	77.4	77.4
N1156	81.6	81.5	80.9
N1157	71.6	71.6	71.5
N1158	51.9	51.8	50.5
N1161	82.7	82.4	82.3
N1162	92.6	92.5	91.8
N1163	86.6	86.5	82.5
N1164	80.5	79.9	78.8
N1165	63	62.6	62.6
N1166	86.7	86.3	86.3
N1167	57.7	57.5	56.9
N1168	39.6	39.4	39.4
N1169	48.8	48.8	48.7
N1170	52.3	52.2	52.2
N1171	54.8	54.7	54.7
N1173	70.9	70.7	69.7
N1174	86.1	85.8	85.8
N1175	82	81.9	81.9
N1191	90.3	90.3	89.5

A total number of 968898 contigs were prepared from clean reads during the MAGs construction process (Table 3), which varies from 95554 (highest) to 16091 (lowest). The number of long contigs ≥5000 bp and very long contigs ≥50000 bp varies from 4621 (highest) to 747 (lowest) and 316 (highest) to 51 (lowest), respectively, which indicates the high quality and depth of the sequencing reads, as well as the effectiveness of the assembler. To increase the accuracy of binning and construction of MAGs, we have excluded the contigs ≲2,500 bp to avoid high contamination and low completeness. The MAGs were validated following the standards defined by the Minimum Information about a Metagenome-Assembled Genome (MIMAG) of bacteria and archaea consortium^43^. In brief, CheckM v1.1.3^31^ was used to calculate the completeness and contamination of each MAG using CheckM marker gene list. Only the high-quality MAGs (completeness >90% and contamination <5%) showing single-copy genes within a phylogenetic lineage^31^ were considered and deposited in the NCBI genome database (BioProject number PRJNA970820). Furthermore, the quality of *Akkermansia* MAGs was assessed considering the type strain of *A. muciniphila* (ATCC BAA-835) (Table 4). The lower number of contigs (varies from 39 to 14), along with scaffold-gap at extensive misassemblies (0) and a number of uncalled bases or N’s (0), confirmed the accuracy of assembly and draft genome quality.

**Table 3:** Assembly statistics of each data set assessed through MetaQuast.

Sample ID	N50	Contigs	Contigs (≥1000 bp)	Contigs (≥5000 bp)	Contigs (≥50000 bp)	Largest contigs	Total length
N1153	6227	48041	18566	2489	258	746854	105729105
N1154	9909	45466	15502	2647	254	564156	103691738
N1155	8925	47210	21537	3309	316	430385	124325910
N1156	5041	33399	10654	1195	140	494532	62771082
N1157	6482	49291	16145	2586	203	658334	101152500
N1158	6622	60260	26331	4621	254	344279	144792077
N1161	3570	51603	16760	2121	158	426442	91504015
N1162	3324	38157	12526	1298	124	622898	67759276
N1163	10804	36485	17629	2633	268	551962	101588829
N1164	2291	69590	21598	1771	209	432752	111061798
N1165	7099	32105	13663	2319	142	539170	76610458
N1166	11262	23907	10892	1786	169	916721	67927401
N1167	13388	28346	8822	957	179	1001980	60863485
N1168	5012	31015	12414	2033	90	305683	64411737
N1169	2728	95554	32177	3148	256	518380	162005710
N1170	7415	73343	26590	4400	353	690106	163873475
N1171	2931	71052	27683	2504	230	416246	129883700
N1173	3562	48745	18083	2057	146	444211	91532050
N1174	3557	16091	6867	747	51	368322	31960148
N1175	9015	20302	9222	1596	136	383053	54938244
N1191	2642	48936	18965	2083	99	503892	83493951

**Table 4:** Genome statistics of *Akkermansia* MAGs taking *A. muciniphila* ATCC BAA-835 as reference.

Samples bin ID	N50	Contigs	Contigs (≥5000 bp)	scaffold gap ext. mis.	GC (%)	N’s
N1157.9	124433	37	31	0	55.41	0
N1164.13	113582	36	36	0	55.59	0
N1167.9	177866	39	30	0	55.84	0
N1170.24	177913	26	23	0	55.34	0
N1173.3	227844	33	24	0	58.13	0
N1174.3	179887	39	33	0	55.96	0
N1175.9	110625	41	41	0	56.8	0
N1191.18	113537	14	13	0	55.6	0

## Supplementary information

Supplementary Information

## Data Availability

We used all open-source software or packages to analyze our data and did not use any custom codes. The version of each package was provided with non-default parameters when required.
