# Supplementary material for: Deep sequencing-derived Metagenome Assembled Genomes from the gut microbiome of liver transplant patients
Source: Sci Data. 2025 Jan 9;12:39. doi: 10.1038/s41597-024-04153-8 (PMC11717916; doi:10.1038/s41597-024-04153-8)
Supplement: Supplementary file 1 — Supplementary Information [file 41597_2024_4153_MOESM1_ESM.pdf]

## Supplementary Information

# Deep sequencing-derived Metagenome Assembled Genomes from the gut microbiome of liver transplant patients

Goutam Banerjee[1], Suraya Rahman Papri[1], Hai Huang[2], Sanjaya Kumar Satapathy[3*], Pratik Banerjee[1*]

1. Department of Food Science and Human Nutrition, University of Illinois at Urbana11 Champaign, Urbana, IL 61801, USA

2. The Feinstein Institutes for Medical Research, Northwell Health, Manhasset, NY, United States

3. Division of Hepatology, Sandra Atlas Bass Center for Liver Diseases & Transplantation, Donald and Barbara Zucker School of Medicine at Hofstra/NorthwellHealth, Manhasset, NY, United States

## Table of Contents

| Table/Fig No. | Descriptions |
|---|---|
| Table S1 | Patient-level demographic and clinical data at the time of stool sample collection |
| Table S2 | The alpha diversity of LT samples estimated by two common diversity indices |
| Table S3 | The completeness and contamination of total 219 high quality MAGs |
| Table S4 | The relative abundance of MAGs in each data set |
| Figure S1 | Differential abundance of three key phyla among different NAS groups |

## Supplementary Information

**Table S1:** Patient-level demographic and clinical data at the time of stool sample collection.

| Patient (sample) ID | Gender | Age | Years from LT | MASLD | NAS |
|---|---|---|---|---|---|
| N1153 | M | 65 | 1 | 0 | 0 |
| N1154 | F | 57 | 1 | 1 | 1 |
| N1155 | F | 58 | 6 | 1 | 5 |
| N1156 | M | 49 | 1 | 0 | 1 |
| N1157 | M | 56 | 1 | 1 | 2 |
| N1158 | F | 69 | 3 | 1 | 5 |
| N1161 | F | 67 | 2 | 1 | 3 |
| N1162 | M | 64 | 1 | 1 | 3 |
| N1163 | F | 67 | 2 | 1 | 4 |
| N1164 | M | 70 | 5 | 1 | 1 |
| N1165 | F | 66 | 4 | 1 | 5 |
| N1166 | F | 51 | 3 | 1 | 3 |
| N1167 | M | 56 | 2 | 1 | 6 |
| N1168 | M | 48 | 1 | 1 | 1 |
| N1169 | M | 66 | 4 | 1 | 2 |
| N1170 | F | 71 | 3 | 1 | 4 |
| N1171 | M | 65 | 7 | 0 | 0 |
| N1173 | M | 65 | 1 | 0 | 0 |
| N1174 | M | 73 | 8 | 1 | 3 |
| N1175 | F | 71 | 3 | 0 | 0 |
| N1191 | **F** | 59 | 6 | 0 | 1 |

**Table S2:** The alpha diversity of LT samples estimated by two common diversity indices.

| Sample ID | Berger Parker's | Simpson's |
|---|---|---|
| N1153 | 0.17923 | 0.9315 |
| N1154 | 0.17923 | 0.9315 |
| N1155 | 0.38627 | 0.821 |
| N1156 | 0.50674 | 0.68476 |
| N1157 | 0.22601 | 0.88095 |
| N1158 | 0.26606 | 0.89682 |
| N1161 | 0.64889 | 0.57263 |
| N1162 | 0.34406 | 0.7665 |
| N1163 | 0.16557 | 0.92355 |
| N1164 | 0.60842 | 0.62081 |
| N1165 | 0.26837 | 0.88239 |
| N1166 | 0.39028 | 0.82495 |
| N1167 | 0.38029 | 0.7623 |
| N1168 | 0.09781 | 0.95689 |
| N1169 | 0.19855 | 0.94229 |
| N1170 | 0.37164 | 0.84607 |
| N1171 | 0.22865 | 0.90037 |
| N1173 | 0.44123 | 0.68477 |
| N1174 | 0.72495 | 0.43669 |
| N1175 | 0.68508 | 0.51626 |
| N1191 | 0.78571 | 0.3798 |

**Table S3:** The completeness and contamination of total 219 high quality MAGs.

| **Metagenome assembled genome (MAGs) with sample ID** | **Completeness (%)** | **Contamination (%)** |
|---|---|---|
| Oscillospiraceae_N1154.9 | 100 | 0.14 |
| *Proteus_mirabilis*_N1162.4 | 100 | 0 |
| Methanobrevibacter_smithii_N1173.2 | 100 | 0 |
| *Megasphaera*_N1156.1 | 100 | 0.6 |
| *Megasphaera*_N1153.15 | 100 | 0.4 |
| *Laribacter_hongkongensis*_N1166.2 | 100 | 0.54 |
| *Fusobacterium_ulcerans*_N1166.6 | 100 | 2.25 |
| *Bilophila_wadsworthia*_N1167.5 | 100 | 0.59 |
| *Bifidobacterium_longum*_N1168.11 | 100 | 0 |
| *Bifidobacterium_longum*_N1161.4 | 100 | 0 |
| *Acidaminococcus_intestini*_N1155.5 | 100 | 0 |
| *Phascolarctobacterium faecium*_N1165.7 | 99.98 | 1.5 |
| *Megasphaera*_N1165.12 | 99.88 | 0.6 |
| *Pseudomonas_aeruginosa*_N1163.10 | 99.68 | 0.11 |
| *Escherichia_coli*_N1167.8 | 99.67 | 0.48 |
| *Escherichia_coli*_N1165.14 | 99.67 | 3.81 |
| *Escherichia_coli*_N1164.9 | 99.67 | 3.5 |
| *Escherichia_coli*_N1156.2 | 99.67 | 0.39 |
| *Collinsella*_N1155.20 | 99.63 | 1.61 |
| *Escherichia_coli*_N1174.2 | 99.62 | 0.15 |
| *Escherichia_coli*_N1161.2 | 99.62 | 0.26 |
| *Escherichia_coli*_N1175.5 | 99.62 | 0.65 |
| Rikenellaceae_N1169.12 | 99.52 | 0.64 |
| *Eubacterium_rectale*_N1173.20 | 99.52 | 0.24 |
| *Eubacterium_rectale*_N1153.19 | 99.52 | 0 |
| *Bacteroides_ovatus*_N1155.13 | 99.51 | 0.04 |
| *Bacteroides_xylanisolvens*_N1156.6 | 99.46 | 0 |
| *Dysgonomonas*_N1175.7 | 99.45 | 3.28 |
| *Phocaeicola dorei* _N1165.11 | 99.44 | 1.5 |
| *Parabacteroides_distasonis*_N1170.16 | 99.42 | 0 |
| *Ruminococcus_torques*_N1169.17 | 99.42 | 0 |
| *Ruminococcus_torques*_N1154.4 | 99.42 | 0 |
| *Bilophila_wadsworthia*_N1156.5 | 99.41 | 0.11 |
| *Megasphaera*_N1191.6 | 99.4 | 0 |
| *Acidaminococcus_intestini*_N1162.2 | 99.4 | 0 |
| *Phascolarctobacterium faecium*_N1153.9 | 99.38 | 1.5 |
| *Escherichia_coli*_N1162.8 | 99.37 | 0.15 |
| *Parasutterella_excrementihominis*_N1169.9 | 99.37 | 0.11 |
| *Blautia_obeum*_N1166.13 | 99.37 | 0.21 |

## Supplementary Information

| **Metagenome assembled genome (MAGs) with sample ID** | **Completeness (%)** | **Contamination (%)** |
|---|---|---|
| *Blautia_obeum*_N1161.8 | 99.37 | 0 |
| *Blautia_obeum*_N1155.8 | 99.37 | 0 |
| *Clostridium_leptum*_N1154.8 | 99.33 | 0 |
| Oscillospiraceae _N1175.8 | 99.32 | 0.68 |
| Acidaminococcaceae_N1164.8 | 99.28 | 2.1 |
| *Alistipes_finegoldii*_N1153.12 | 99.28 | 0.48 |
| *Bacteroides_stercoris*_N1156.3 | 99.26 | 0 |
| *Bacteroides_fragilis*_N1167.13 | 99.26 | 0 |
| *Parabacteroides_merdae*_N1162.1 | 99.25 | 1.53 |
| *Phocaeicola vulgatus* _N1158.23 | 99.25 | 0 |
| *Hafnia_paralvei*_N1164.7 | 99.24 | 0.7 |
| *Parabacteroides_merdae*_N1154.6 | 99.23 | 0.51 |
| *Bacteroides_dorei*_N1154.10 | 99.23 | 0.85 |
| Bacillota _N1167.4 | 99.22 | 1.48 |
| *Eggerthella_lenta*_N1168.12 | 99.19 | 0 |
| *Collinsella_aerofaciens*_N1173.11 | 99.19 | 1.61 |
| *Bacteroides*_N1166.4 | 99.19 | 0.33 |
| *Limosilactobacillus fermentum* _N1170.28 | 99.18 | 0 |
| *Bacteroides_dorei*_N1169.20 | 99.06 | 1.5 |
| Lachnospiraceae_N1167.6 | 99.05 | 0 |
| Lachnospiraceae_N1169.13 | 99.03 | 1.71 |
| Lachnospiraceae_N1171.17 | 99.02 | 0 |
| *Bifidobacterium_longum*_N1173.10 | 99.01 | 0.55 |
| *Streptococcus_salivarius*_N1170.6 | 98.92 | 1.49 |
| *Limosilactobacillus fermentum* _N1168.2 | 98.91 | 1.2 |
| *Bacteroides_fragilis*_N1157.4 | 98.88 | 0.93 |
| *Oligella_urethralis*_N1163.6 | 98.88 | 0 |
| Fusobacteriaceae_N1165.6 | 98.88 | 2.25 |
| *Phocaeicola vulgatus*_N1153.13 | 98.87 | 0.4 |
| *Ruminococcus_torques*_N1166.11 | 98.83 | 0.29 |
| *Ruminococcus_torques*_N1158.3 | 98.83 | 0.74 |
| *Bifidobacterium_longum*_N1170.9 | 98.82 | 2.59 |
| *Bacteroides_ovatus*_N1154.5 | 98.81 | 0.16 |
| *Alistipes_finegoldii*_N1164.6 | 98.8 | 0.48 |
| Lachnospiraceae_N1164.3 | 98.78 | 0 |
| *Blautia_obeum*_N1154.19 | 98.73 | 0 |
| *Ruminococcus_bromii*_N1169.23 | 98.66 | 1.01 |
| Bacillota _N1157.17 | 98.66 | 0 |
| Oscillospiraceae _N1158.12 | 98.64 | 0.68 |
| *Faecalibacterium_prausnitzii*_N1158.17 | 98.64 | 0 |
| *Prevotella_buccae*_N1167.1 | 98.64 | 0.34 |

# Supplementary Information

| **Metagenome assembled genome (MAGs) with sample ID** | **Completeness (%)** | **Contamination (%)** |
|---|---|---|
| *Anaerostipes_hadrus*_N1170.23 | 98.52 | 2.35 |
| *Ruthenibacterium_lactatiformans*_N1158.15 | 98.52 | 0 |
| *Blautia_obeum*_N1153.3 | 98.33 | 0.63 |
| *Fusicatenibacter_saccharivorans*_N1153.7 | 98.31 | 0.65 |
| *Ruminococcus_bromii*_N1173.14 | 98.28 | 9.48 |
| *Collinsella*_N1154.15 | 98.19 | 1.09 |
| *Bacteroides_vulgatus*_N1163.8 | 98.16 | 0 |
| *Bacteroides_uniformis*_N1164.4 | 98.14 | 0.19 |
| *Dialister_invisus*_N1170.8 | 98.1 | 0.63 |
| *Dialister_invisus*_N1167.7 | 98.1 | 1.92 |
| *Dialister_invisus*_N1156.8 | 98.1 | 0.63 |
| *Blautia_obeum*_N1173.21 | 98.1 | 1.11 |
| *Bacteroides_fragilis*_N1162.6 | 98.1 | 0 |
| *Ruminococcus_bromii*_N1170.3 | 97.99 | 0.67 |
| Oscillospiraceae _N1170.17 | 97.99 | 1.68 |
| Oscillospiraceae _N1158.9 | 97.99 | 0.34 |
| Oscillospiraceae _N1157.1 | 97.99 | 0 |
| Bacillota _N1164.14 | 97.99 | 1.34 |
| Bacillota _N1161.10 | 97.99 | 0 |
| *Clostridium_leptum*_N1155.7 | 97.99 | 1.34 |
| *Blautia*_N1171.6 | 97.99 | 2.01 |
| *Faecalibacterium_prausnitzii*_N1155.22 | 97.96 | 0 |
| *Bacteroides_caccae*_N1161.1 | 97.96 | 0 |
| *Akkermansia_muciniphila*_N1173.3 | 97.96 | 0.68 |
| *Akkermansia_muciniphila*_N1170.24 | 97.96 | 0 |
| *Akkermansia_muciniphila*_N1167.9 | 97.96 | 0 |
| *Akkermansia_muciniphila*_N1164.13 | 97.96 | 0.68 |
| *Akkermansia_muciniphila*_N1157.9 | 97.96 | 0 |
| *Akkermansia*_N1175.9 | 97.96 | 0 |
| *Akkermansia*_N1174.3 | 97.96 | 0.68 |
| *Ruminococcus_gnavus*_N1154.12 | 97.95 | 0.58 |
| Bacillota _N1170.38 | 97.95 | 2.64 |
| *Parabacteroides_distasonis*_N1155.19 | 97.93 | 1.73 |
| *Enterocloster bolteae*_N1154.17 | 97.79 | 0.68 |
| *Bacteroides_uniformis*_N1157.12 | 97.79 | 0.99 |
| Oscillospiraceae _N1175.6 | 97.77 | 1.42 |
| *Monoglobus_pectinilyticus*_N1170.19 | 97.76 | 0.34 |
| *Bacteroides_uniformis*_N1171.14 | 97.74 | 0.12 |
| *Citrobacter_freundii*_N1163.9 | 97.72 | 1.24 |
| *Parabacteroides_distasonis*_N1157.2 | 97.7 | 0.77 |
| *Blautia_obeum*_N1156.9 | 97.67 | 0.02 |

# Supplementary Information

| **Metagenome assembled genome (MAGs) with sample ID** | **Completeness (%)** | **Contamination (%)** |
|---|---|---|
| *Bacteroides intestinalis*_N1163.1 | 97.67 | 0.75 |
| *Ruminococcus_bromii*_N1158.13 | 97.65 | 1.01 |
| Bacillota _N1170.15 | 97.65 | 0.67 |
| *Eubacterium_siraeum*_N1169.1 | 97.65 | 0.34 |
| *Clostridium_bolteae*_N1158.18 | 97.63 | 0.11 |
| *Acidaminococcus_intestini*_N1191.4 | 97.6 | 0 |
| *Collinsella_intestinalis*_N1153.5 | 97.58 | 0.81 |
| *Parabacteroides_distasonis*_N1171.13 | 97.55 | 0.77 |
| *Bacteroides_uniformis*_N1153.20 | 97.51 | 0 |
| *Parabacteroides_distasonis*_N1169.6 | 97.5 | 1.06 |
| *Phascolarctobacterium_succinatutens*_N1169.7 | 97.45 | 1.8 |
| Bacteroidaceae_N1166.5 | 97.4 | 1.05 |
| *Ruminococcus_bromii*_N1168.8 | 97.32 | 1.34 |
| Oscillospiraceae _N1169.2 | 97.32 | 0.67 |
| Oscillospiraceae _N1157.15 | 97.32 | 0.67 |
| *Flavonifractor_plautii*_N1154.2 | 97.32 | 2.48 |
| Bacillota_N1173.4 | 97.32 | 1.34 |
| Bacillota_N1170.14 | 97.32 | 0 |
| Bacillota_N1155.18 | 97.32 | 1.34 |
| *Prevotella_copri*_N1169.11 | 97.3 | 0.84 |
| *Ruthenibacterium_lactatiformans*_N1170.7 | 97.28 | 0.34 |
| *Gemmiger_formicilis*_N1153.8 | 97.28 | 0.82 |
| | | |
| *Faecalibacterium_prausnitzii*_N1167.10 | 97.28 | 0 |
| *Faecalibacterium_prausnitzii*_N1157.5 | 97.25 | 0 |
| *Roseburia_faecis*_N1169.18 | 97.18 | 1.45 |
| Bacillota _N1158.14 | 97.15 | 3.15 |
| *Blautia_obeum*_N1168.5 | 96.92 | 0 |
| Oscillospiraceae _N1170.4 | 96.91 | 1.36 |
| Lachnospiraceae_N1170.2 | 96.84 | 1.41 |
| *Citrobacter_koseri*_N1155.12 | 96.83 | 3.56 |
| *Parabacteroides_merdae*_N1155.11 | 96.82 | 1.15 |
| *Ruminococcus_torques*_N1163.11 | 96.78 | 0.7 |
| Lachnospiraceae_N1154.11 | 96.72 | 0 |
| *Ruminococcus_bromii*_N1171.1 | 96.64 | 0.67 |
| Bacillota _N1158.5 | 96.64 | 1.01 |
| *Barnesiella_intestinihominis*_N1169.5 | 96.6 | 0 |
| *Gemmiger_formicilis*_N1163.2 | 96.6 | 0.02 |
| Bacillota _N1169.16 | 96.55 | 0.19 |
| *Roseburia_hominis*_N1158.16 | 96.38 | 0.48 |
| *Roseburia_intestinalis*_N1157.13 | 96.38 | 1.33 |

| **Metagenome assembled genome (MAGs) with sample ID** | **Completeness (%)** | **Contamination (%)** |
|---|---|---|
| *Parabacteroides_distasonis*_N1158.8 | 96.35 | 1.86 |
| Bacillota_N1166.7 | 96.31 | 2.51 |
| Blautia_N1153.11 | 96.27 | 0.32 |
| *Ruminococcus_bicirculans*_N1170.34 | 96.24 | 0.23 |
| *Holdemanella*_N1154.3 | 96.23 | 0.71 |
| Pseudomonadota _N1163.16 | 96.09 | 2 |
| *Bacteroides_caccae*_N1191.7 | 96.08 | 3.18 |
| *Bacteroides_caccae*_N1161.11 | 96.05 | 1.04 |
| *Parabacteroides_distasonis*_N1163.7 | 95.99 | 1.67 |
| Pseudomonadota _N1164.5 | 95.7 | 0 |
| *Ruminococcus_torques*_N1164.15 | 95.62 | 3.19 |
| *Flavonifractor_plautii*_N1157.3 | 95.61 | 1.48 |
| *Ruminococcus_torques*_N1171.5 | 95.32 | 0 |
| *Anaerotignum_lactatifermentans*_N1155.9 | 95.3 | 0.08 |
| *Anaerotignum*_N1164.1 | 95.3 | 0 |
| *Phocaeicola vulgatus* _N1191.2 | 95.11 | 0.56 |
| *Blautia*_N1161.12 | 94.96 | 0.02 |
| *Bilophila_wadsworthia*_N1163.4 | 94.84 | 1.28 |
| *Clostridium*_N1158.21 | 94.63 | 1.63 |
| Oscillospiraceae _N1169.15 | 94.63 | 0.9 |
| Pseudomonadota_N1170.29 | 94.62 | 1.24 |
| *Escherichia_coli*_N1173.17 | 94.61 | 0.1 |
| Bacteroidetes_N1169.19 | 94.49 | 0.56 |
| Bacillota _N1170.26 | 94.41 | 1.01 |
| Bacillota _N1157.18 | 94.38 | 0.53 |
| Bacteroidetes_N1169.14 | 94.18 | 0.75 |
| *Ruthenibacterium_lactatiformans*_N1164.2 | 94.01 | 0 |
| *Ruminococcus_torques*_N1161.5 | 93.82 | 0 |
| *Ruminococcus_torques*_N1168.1 | 93.29 | 0.63 |
| *Blautia_obeum*_N1165.9 | 93.12 | 0.02 |
| Oscillospiraceae _N1170.21 | 93.11 | 1.34 |
| Oscillospiraceae _N1173.1 | 92.95 | 3.08 |
| *Rum*inococcus_N1154.16 | 92.87 | 1.2 |
| Lachnospiraceae_N1171.8 | 92.87 | 0.88 |
| *Parabacteroides_distasonis*_N1154.14 | 92.74 | 0.77 |
| Oscillospiraceae _N1170.18 | 92.62 | 0 |
| Oscillospiraceae _N1158.22 | 92.62 | 1.01 |
| *Ruthenibacterium_lactatiformans*_N1157.19 | 92.17 | 0.5 |
| *Roseburia*_N1157.16 | 92.15 | 2.52 |
| *Phascolarctobacterium_succinatutens*_N1191.10 | 92.14 | 1.95 |
| *Clostridium_leptum*_N1170.10 | 92.09 | 2.85 |

# Supplementary Information

| **Metagenome assembled genome (MAGs) with sample ID** | **Completeness (%)** | **Contamination (%)** |
|---|---|---|
| *Clostridium*_N1161.6 | 91.64 | 2.32 |
| *Escherichia_coli*_N1166.10 | 91.55 | 0.45 |
| Bacillota _N1170.22 | 91.49 | 0.71 |
| *Roseburia_inulinivorans*_N1156.7 | 91.46 | 1.04 |
| Bacillota _N1171.4 | 91.42 | 1.01 |
| *Bifidobacterium_longum*_N1153.4 | 91.27 | 1.83 |
| *Gemmiger_formicilis*_N1165.4 | 91.15 | 0.85 |
| Lachnospiraceae_N1171.2 | 90.9 | 0.21 |
| *Parabacteroides_merdae*_N1162.11 | 90.89 | 0.64 |
| *Bacteroides_ovatus*_N1158.6 | 90.87 | 2.07 |
| Bacillota _N1174.1 | 90.83 | 1.07 |
| *Anaerostipes_hadrus*_N1168.10 | 90.66 | 4.61 |
| *Escherichia_coli*_N1155.6 | 90.44 | 3.37 |
| *Bacteroides_finegoldii*_N1170.30 | 90.43 | 0.63 |
| *Coprococcus*_N1153.14 | 90.07 | 0.63 |
| *Collinsella_aerofaciens*_N1165.2 | 89.15 | 0.95 |
| *Akkermansia_muciniphila*_N1191.18 | 94.69 | 0 |

**Table S4:** The relative abundance of MAGs in each data set.

| Sample ID | Organism | Relative Abundance (%) |
|---|---|---|
| N1153 | *Blautia_obeum*_N1153.3 | 32.9 |
| | *Bifidobacterium_longum*_N1153.4 | 0.6 |
| | *Collinsella_intestinalis*_N1153.5 | 0.7 |
| | *Fusicatenibacter_saccharivorans*_N1153.7 | 4.58 |
| | *Gemmiger_formicilis*_N1153.8 | 3.07 |
| | *Phascolarctobacterium faecium_* _N1153.9 | 3.91 |
| | *Blautia*_N1153.11 | 7.8 |
| | *Alistipes_finegoldii*_N1153.12 | 2.01 |
| | *Bacteroides_vulgatus*_N1153.13 | 14.19 |
| | *Coprococcus*_N1153.14 | 2.67 |
| | *Megasphaera*_N1153.15 | 1.23 |
| | *Eubacterium_rectale*_N1153.19 | 3.75 |
| | *Bacteroides_uniformis*_N1153.20 | 3.97 |
| | | |
| N1154 | *Flavonifractor_plautii*_N1154.2 | 1.88 |
| | *Holdemanella*_N1154.3 | 2.39 |
| | *Ruminococcus_torques*_N1154.4 | 4.11 |
| | *Bacteroides_ovatus*_N1154.5 | 15.67 |
| | *Parabacteroides_merdae*_N1154.6 | 4.71 |
| | *Clostridium_leptum*_N1154.8 | 2.45 |
| | Ruminococcaceae_N1154.9 | 1.51 |
| | *Bacteroides_dorei*_N1154.10 | 9.1 |
| | Lachnospiraceae_N1154.11 | 2.92 |
| | *Ruminococcus_gnavus*_N1154.12 | 3.88 |
| | *Parabacteroides_distasonis*_N1154.14 | 2.81 |
| | *Collinsella*_N1154.15 | 0.61 |
| | Ruminococcus_N1154.16 | 2.29 |
| | *Clostridium_bolteae*_N1154.17 | 3.66 |
| | *Blautia_obeum*_N1154.19 | 41.46 |
| | | |
| N1155 | *Acidaminococcus_intestini*_N1155.5 | 10.42 |
| | *Escherichia_coli*_N1155.6 | 5.81 |
| | *Clostridium_leptum*_N1155.7 | 2.43 |
| | *Blautia_obeum*_N1155.8 | 11.41 |
| | *Anaerotignum_lactatifermentans*_N1155.9 | 1.26 |
| | *Parabacteroides_merdae*_N1155.11 | 3.13 |
| | *Citrobacter_koseri*_N1155.12 | 5.4 |
| | *Bacteroides_ovatus*_N1155.13 | 36.58 |
| | Bacillota _N1155.18 | 2.23 |
| | *Parabacteroides_distasonis*_N1155.19 | 2.32 |
| | *Collinsella*_N1155.20 | 1.61 |
| | *Faecalibacterium_prausnitzii*_N1155.22 | 1.6 |

# Supplementary Information

| Sample ID | Organism | Relative Abundance (%) |
|---|---|---|
| | | |
| N1156 | *Megasphaera*_N1156.1 | 2.77 |
| | *Escherichia_coli*_N1156.2 | 41.51 |
| | *Bacteroides_stercoris*_N1156.3 | 20.85 |
| | *Bilophila_wadsworthia*_N1156.5 | 2.34 |
| | *Bacteroides_xylanisolvens*_N1156.6 | 11.09 |
| | *Roseburia_inulinivorans*_N1156.7 | 1.89 |
| | *Dialister_invisus*_N1156.8 | 8 |
| | *Blautia_obeum*_N1156.9 | 3.89 |
| | | |
| N1157 | Ruminococcaceae_N1157.1 | 6.26 |
| | *Parabacteroides_distasonis*_N1157.2 | 7.23 |
| | *Flavonifractor_plautii*_N1157.3 | 3.66 |
| | *Bacteroides_fragilis*_N1157.4 | 16.61 |
| | *Faecalibacterium_prausnitzii*_N1157.5 | 2.82 |
| | *Akkermansia_muciniphila*_N1157.9 | 11.64 |
| | *Bacteroides_uniformis*_N1157.12 | 15.16 |
| | *Roseburia_intestinalis*_N1157.13 | 7.99 |
| | Ruminococcaceae_N1157.15 | 9.71 |
| | *Roseburia*_N1157.16 | 1.9 |
| | Bacillota _N1157.17 | 3.36 |
| | Bacillota _N1157.18 | 2.22 |
| | *Ruthenibacterium_lactatiformans*_N1157.19 | 2.11 |
| | | |
| N1158 | *Ruminococcus_torques*_N1158.3 | 4.8 |
| | Bacillota _N1158.5 | 2.06 |
| | *Bacteroides_ovatus*_N1158.6 | 4.5 |
| | *Parabacteroides_distasonis*_N1158.8 | 2.3 |
| | Oscillospiraceae _N1158.9 | 14.56 |
| | Oscillospiraceae _N1158.12 | 9.42 |
| | *Ruminococcus_bromii*_N1158.13 | 1.73 |
| | Bacillota _N1158.14 | 1.83 |
| | *Ruthenibacterium_lactatiformans*_N1158.15 | 3.89 |
| | *Roseburia_hominis*_N1158.16 | 3.13 |
| | *Faecalibacterium_prausnitzii*_N1158.17 | 6.97 |
| | *Clostridium_bolteae*_N1158.18 | 4.13 |
| | *Clostridium*_N1158.21 | 4.19 |
| | Oscillospiraceae _N1158.22 | 3.5 |
| | *Phocaeicola vulgatus* _N1158.23 | 15.11 |
| | | |
| N1161 | *Bacteroides_caccae*_N1161.1 | 4.93 |
| | *Escherichia_coli*_N1161.2 | 55.97 |
| | *Bifidobacterium_longum*_N1161.4 | 1.56 |

## Supplementary Information

| Sample ID | Organism | Relative Abundance (%) |
|---|---|---|
| | *Ruminococcus_torques*_N1161.5 | 2.03 |
| | *Clostridium*_N1161.6 | 2.83 |
| | *Blautia_obeum*_N1161.8 | 6.72 |
| | Bacillota _N1161.10 | 1.98 |
| | *Bacteroides_caccae*_N1161.11 | 3.5 |
| | *Blautia*_N1161.12 | 1.84 |
| | | |
| N1162 | *Parabacteroides_merdae*_N1162.1 | 20.94 |
| | *Acidaminococcus_intestini*_N1162.2 | 1.51 |
| | *Proteus_mirabilis*_N1162.4 | 25.55 |
| | *Bacteroides_fragilis*_N1162.6 | 9.61 |
| | *Escherichia_coli*_N1162.8 | 31.47 |
| | *Parabacteroides_merdae*_N1162.11 | 2.11 |
| | | |
| N1163 | *Bacteroides intestinalis*_N1163.1 | 9.87 |
| | *Gemmiger_formicilis*_N1163.2 | 2.55 |
| | *Bilophila_wadsworthia*_N1163.4 | 1.88 |
| | *Oligella_urethralis*_N1163.6 | 4.32 |
| | *Parabacteroides_distasonis*_N1163.7 | 2.62 |
| | *Bacteroides_vulgatus*_N1163.8 | 11.82 |
| | *Citrobacter_freundii*_N1163.9 | 19.97 |
| | *Pseudomonas_aeruginosa*_N1163.10 | 11.71 |
| | *Ruminococcus_torques*_N1163.11 | 2.07 |
| | Pseudomonadota _N1163.16 | 1.17 |
| | | |
| N1164 | *Anaerotignum*_N1164.1 | 1.41 |
| | *Ruthenibacterium_lactatiformans*_N1164.2 | 2.96 |
| | Lachnospiraceae_N1164.3 | 4.97 |
| | *Bacteroides_uniformis*_N1164.4 | 4.44 |
| | Pseudomonadota_N1164.5 | 1.02 |
| | *Alistipes_finegoldii*_N1164.6 | 3.14 |
| | *Hafnia_paralvei*_N1164.7 | 9.53 |
| | Acidaminococcaceae_N1164.8 | 2.91 |
| | *Escherichia_coli*_N1164.9 | 50.66 |
| | *Akkermansia_muciniphila*_N1164.13 | 4.46 |
| | Bacillota_N1164.14 | 2.21 |
| | *Ruminococcus_torques*_N1164.15 | 2.46 |
| | | |
| N1165 | *Collinsella_aerofaciens*_N1165.2 | 0.79 |
| | *Gemmiger_formicilis*_N1165.4 | 1.11 |
| | Fusobacteriaceae_N1165.6 | 29.43 |
| | *Phascolarctobacterium faecium*_N1165.7 | 3.4 |
| | *Blautia_obeum*_N1165.9 | 9.12 |

## Supplementary Information

| Sample ID | Organism | Relative Abundance (%) |
| --- | --- | --- |
| | *Bacteroides_dorei*_N1165.11 | 10.8 |
| | *Megasphaera*_N1165.12 | 20.27 |
| | *Escherichia_coli*_N1165.14 | 8.3 |
| | | |
| N1166 | *Laribacter_hongkongensis*_N1166.2 | 3.54 |
| | *Bacteroides*_N1166.4 | 20.33 |
| | Bacteroidaceae_N1166.5 | 4.19 |
| | *Fusobacterium_ulcerans*_N1166.6 | 8.01 |
| | Bacillota_N1166.7 | 1.14 |
| | *Escherichia_coli*_N1166.10 | 35.21 |
| | *Ruminococcus_torques*_N1166.11 | 2.14 |
| | *Blautia_obeum*_N1166.13 | 4 |
| | | |
| N1167 | *Prevotella_buccae*_N1167.1 | 2.09 |
| | Bacillota_N1167.4 | 1.57 |
| | *Bilophila_wadsworthia*_N1167.5 | 5.94 |
| | Lachnospiraceae_N1167.6 | 12.14 |
| | *Dialister_invisus*_N1167.7 | 2.27 |
| | *Escherichia_coli*_N1167.8 | 21.45 |
| | *Akkermansia_muciniphila*_N1167.9 | 10.3 |
| | *Faecalibacterium_prausnitzii*_N1167.10 | 2.53 |
| | *Bacteroides_fragilis*_N1167.13 | 14.82 |
| | | |
| N1168 | *Ruminococcus_torques*_N1168.1 | 8.79 |
| | *Lactobacillus_fermentum*_N1168.2 | 1 |
| | *Blautia_obeum*_N1168.5 | 34.36 |
| | *Ruminococcus_bromii*_N1168.8 | 1.74 |
| | *Anaerostipes_hadrus*_N1168.10 | 2.99 |
| | *Bifidobacterium_longum*_N1168.11 | 2.77 |
| | *Eggerthella_lenta*_N1168.12 | 1.8 |
| | | |
| N1169 | *Eubacterium_siraeum*_N1169.1 | 1.87 |
| | Oscillospiraceae_N1169.2 | 4.72 |
| | *Barnesiella_intestinihominis*_N1169.5 | 3.99 |
| | *Parabacteroides_distasonis*_N1169.6 | 2.91 |
| | *Phascolarctobacterium_succinatutens*_N1169.7 | 1.26 |
| | *Parasutterella_excrementihominis*_N1169.9 | 1.78 |
| | *Prevotella_co*pri_N1169.11 | 4.16 |
| | Rikenellaceae_N1169.12 | 5.95 |
| | Lachnospiraceae_N1169.13 | 7.81 |
| | Bacteroidetes_N1169.14 | 1.3 |
| | Oscillospiraceae_N1169.15 | 2.15 |
| | Bacillota_N1169.16 | 2.65 |

| Sample ID | Organism | Relative Abundance (%) |
|---|---|---|
| | *Ruminococcus_torques*_N1169.17 | 6.61 |
| | *Roseburia_faecis*_N1169.18 | 4.29 |
| | Bacteroidetes_N1169.19 | 2.49 |
| | *Bacteroides_dorei*_N1169.20 | 11.04 |
| | *Ruminococcus_bromii*_N1169.23 | 2.34 |
| | | |
| N1170 | Lachnospiraceae_N1170.2 | 4.11 |
| | *Ruminococcus_bromii*_N1170.3 | 2.08 |
| | Oscillospiraceae_N1170.4 | 1.97 |
| | *Streptococcus_salivarius*_N1170.6 | 2.54 |
| | *Ruthenibacterium_lactatiformans*_N1170.7 | 2.46 |
| | *Dialister_invisus*_N1170.8 | 2.77 |
| | *Bifidobacterium_longum*_N1170.9 | 0.88 |
| | *Clostridium_leptum*_N1170.10 | 1.27 |
| | Bacillota_N1170.14 | 1.7 |
| | Bacillota_N1170.15 | 1.24 |
| | *Parabacteroides_distasonis*_N1170.16 | 4.79 |
| | Oscillospiraceae_N1170.17 | 8.06 |
| | Oscillospiraceae_N1170.18 | 2.02 |
| | *Monoglobus_pectinilyticus*_N1170.19 | 2.43 |
| | Oscillospiraceae_N1170.21 | 1.58 |
| | Bacillota_N1170.22 | 0.88 |
| | *Anaerostipes_hadrus*_N1170.23 | 1.88 |
| | *Akkermansia_muciniphila*_N1170.24 | 19.21 |
| | Bacillota_N1170.26 | 0.95 |
| | *Lactobacillus_fermentum*_N1170.28 | 1.05 |
| | Pseudomonadota_N1170.29 | 0.74 |
| | *Bacteroides_finegoldii*_N1170.30 | 3.23 |
| | *Ruminococcus_bicirculans*_N1170.34 | 1.1 |
| | Bacillota_N1170.38 | 2.27 |
| | | |
| N1171 | *Ruminococcus_bromii*_N1171.1 | 1.99 |
| | Lachnospiraceae_N1171.2 | 5.44 |
| | Bacillota_N1171.4 | 1.17 |
| | *Ruminococcus_torques*_N1171.5 | 6.44 |
| | *Blautia*_N1171.6 | 12.41 |
| | Lachnospiraceae_N1171.8 | 2.37 |
| | *Parabacteroides_distasonis*_N1171.13 | 2.6 |
| | *Bacteroides_uniformis*_N1171.14 | 9.44 |
| | Lachnospiraceae_N1171.17 | 3.69 |
| | | |
| N1173 | Oscillospiraceae_N1173.1 | 1.43 |
| | *Methanobrevibacter_smithii*_N1173.2 | 1.29 |

| **Sample ID** | **Organism** | **Relative Abundance (%)** |
|---|---|---|
| | *Akkermansia_muciniphila*_N1173.3 | 25.31 |
| | Bacillota_N1173.4 | 4.99 |
| | Lachnospiraceae_N1173.8 | 1.68 |
| | *Bifidobacterium_longum*_N1173.10 | 1.27 |
| | *Collinsella_aerofaciens*_N1173.11 | 1.43 |
| | *Ruminococcus_bromii*_N1173.14 | 1.4 |
| | *Escherichia_coli*_N1173.17 | 27.25 |
| | *Eubacterium_rectale*_N1173.20 | 3.37 |
| | *Blautia_obeum*_N1173.21 | 9.1 |
| | | |
| N1174 | Bacillota_N1174.1 | 1.31 |
| | *Escherichia_coli*_N1174.2 | 17.55 |
| | *Akkermansia*_N1174.3 | 72.49 |
| | | |
| N1175 | *Escherichia_coli*_N1175.5 | 9.88 |
| | Oscillospiraceae_N1175.6 | 3.1 |
| | *Dysgonomonas*_N1175.7 | 2.12 |
| | Oscillospiraceae_N1175.8 | 16.76 |
| | *Akkermansia*_N1175.9 | 54.38 |
| | | |
| N1191 | *Phocaeicola vulgatus*_N1191.2 | 3.96 |
| | *Acidaminococcus_intestini*_N1191.4 | 1.36 |
| | *Megasphaera*_N1191.6 | 3.18 |
| | *Bacteroides_caccae*_N1191.7 | 3.49 |
| | *Phascolarctobacterium_succinatutens*_N1191.10 | 0.91 |
| | *Akkermansia_muciniphila*_N1191.18 | 35 |

# Supplementary Information

| | NAS (0-2) | | | | | | | | | | | NAS (3-4) | | | | | NAS (>=5) | | | |
|---|---|---|---|---|---|---|---|---|---|---|---|---|---|---|---|---|---|---|---|---|
| **Phylum** | N1153 | N1154 | N1156 | N1157 | N1164 | N1168 | N1169 | N1171 | N1173 | N1175 | N1191 | N1161 | N1162 | N1163 | N1166 | N1174 | N1155 | N1158 | N1165 | N1167 |
| Fusobacteria | 0 | 0.021 | 0.0001 | 0 | 6.4E-05 | 0.0004 | 0 | 0.00029 | 0 | 0 | 0 | 0.00017 | 1.5E-05 | 0 | 0.07924 | 0 | 0.000523 | 0 | 0.1117 | 0.00017 |
| Euryarchaeota | 0 | 1E-04 | 0 | 0 | 0 | 0.0002 | 0.000766 | 0 | 0.014 | 0 | 4.39E-05 | 0 | 0 | 0 | 0 | 0 | 0 | 0.000242 | 0 | 0 |
| Verrucomicrobia | 0 | 0 | 0 | 0.1557 | 0.05263 | 0 | 0.005131 | 0 | 0.347 | 0.687 | 0.7855 | 0.00026 | 0 | 0.00011 | 3.12E-05 | 0.7267 | 7.29E-05 | 0 | 0 | 0.1747 |

Figure S1. Differential abundance of three key phyla among different NAS groups.
